# Physicians in police tactical teams – ethical considerations

**DOI:** 10.1186/s13049-023-01110-z

**Published:** 2023-08-29

**Authors:** Timothee de Valence, Laurent Suppan

**Affiliations:** 1 Department of Anaesthesia, The Royal London Hospital, Barts Health NHS Trust, London, UK; 2 Division of Emergency Medicine, Department of Anaesthesiology, Pharmacology, Intensive Care and Emergency Medicine, Geneva University Hospitals, Geneva, Switzerland

**Keywords:** Damage control, Ethics, Police tactical team, Prehospital, Tactical medicine

## Abstract

High-profile mass shootings, terrorist attacks, and experience acquired during recent conflicts have led to a shift in police tactics, who now follow an aggressive approach to immediately neutralize the threat in addition to providing early tactical medical care. A growing number of police tactical teams now include physicians in their ranks to increase the level of forward care. Many ethical questions arise from having physicians on police tactical teams, such as the notion of risk, the use of force, and the ultimate role the physician is expected to play. Having a physician in such a team may be an invaluable asset to increase the team’s safety and allow for advanced forward care, however, this requires two important conditions. The first is that the role of the physician is clearly defined and that what is expected of him is in line with medical ethics, while the second is extensive tactical training with the team to collaborate flawlessly in this complex, high-stress environment.

In the wake of Columbine and other high-profile mass shootings, law enforcement agencies shifted toward an approach known as “rapid deployment” [1]. This involves training officers to immediately enter the scene of an active shooter situation and engage the shooter, aiming to neutralize the threat as soon as possible [2]. In addition to early threat elimination, concurrent immediate medical care in the “hot zone” has become the standard of care in tactical operations. Courses such as Tactical Emergency Casualty Care (TECC) have enabled forward operators to acquire essential skills, allowing them to provide simple, life-saving interventions [3]. Among these, hemorrhage control is of paramount importance [4]. There have been many ways that emergency care under fire has been implemented, with some services training police officers to provide first aid, while others have chosen to embed EMTs, paramedics, and even physicians in police tactical teams [5, 6]. While doctors have seen frontline action for well over a century in the military setting, integrating physicians into civilian police tactical teams is somewhat recent. Tactical medicine has even evolved to become a subspecialty in itself, with many universities in the United States offering dedicated fellowship programs [7, 8]. While the addition of a physician in police tactical teams might seem a positive approach, allowing for the delivery of advanced forward care in the “hot zone” which is usually out of bounds to other medical professionals, this also raises many ethical questions.

Risk: Working in a tactical setting is a high-risk activity, and doctors embedded in police tactical teams may be put in situations that pose a risk to their safety. While physicians may provide invaluable advanced care in the field, the risks they are exposed to must be carefully weighed against any potential benefit they may offer [9]. Depending on the circumstances, physicians might need to be part of the forward assault team, which implies maximum exposure and risk, while other systems might place tactical physicians in moderate to low-risk areas (“warm” and “cold” zones). The choice of placing physicians in forward positions or delegating forward medical tasks to other providers (EMTs, paramedics, or even police officers) should be decided in advance depending on local needs and environment types. The tactical physician must not be an add-on that slows or hampers the team but rather a high-performing member of the tactical team. It is therefore crucial for doctors who work on police tactical teams to receive specialized training to be able to function effectively within a high-stress and fast-paced environment and to synchronize perfectly with the team and its objectives.

Use of force: When physicians are part of a tactical police team, they may be involved in the use of force against individuals, especially if they are armed, as is the case in some agencies. It is important to ensure that physicians are trained in the appropriate use of force and that their actions are consistent with medical ethics. On the other hand, one could argue that the elimination of the threat, which is the main role of a police tactical team, might be equivalent to saving lives, as leaving the threat unopposed would certainly have resulted in excess injury and death. While harm might be unavoidable to accomplish this goal, it is for the greater good. Moreover, some tactical physicians have noted that their presence in the police tactical team might have a harm-reducing effect [10]. Indeed, having a physician on the team might increase the other members’ sense of safety by having immediate access to medical attention, if required. This, by reducing the team members’ level of stress, may mitigate the risk of an accidental, negligent, or unintentional firearm discharge.

Duty to the patient vs. duty to the team: Physicians have a duty to their patients, to whom they should provide the best possible care. However, when a physician is a tactical police team member, they also have a duty to the team and its objectives. A loyalty issue may arise when physicians are also sworn police officers. This can create a conflict of interest, as the physician may be required to prioritize the needs of the team over the needs of the patient. Furthermore, in situations where team members are wounded, implicit bias might influence the physician to prioritize care for members of the team at the expense of non-police victims.

The role of the physician: The role of a physician on a tactical police team may not always be clear. Physicians are trained to provide medical care and may not have the same level of training or expertise in tactical operations as other team members. It is important to ensure that physicians understand their roles and responsibilities on the team. While the decision concerning medical care must solely be the decision of the physician, tactical decisions such as emergency evacuation or declaring an area as being “safe” must be the responsibility of the police, and physicians must abide by these orders.

Perception of impartiality: The presence of doctors on police tactical teams may create the perception that medical professionals are aligned with law enforcement agencies, which can undermine public trust in the impartiality of the medical profession. Suspects might also be less likely to ask for or accept care from a physician embedded with law enforcement, and doctors have to make it clear that the care they provide is confidential and independent of their role as police physicians. Wearing a different uniform with clear identification might mitigate the negative association one may make when engaging with the physician, but might also jeopardize their safety by singling them out. Another example of an ethical issue regarding the perception of impartiality is the role of police physicians in the forced return of migrants [11]. Professional independence might be difficult to maintain when physicians are requested to assist with forced repatriations of migrants, where decision-making may be biased as the physician is mandated by the police and is required to enable a transfer. Does sedating an agitated migrant for a forced repatriation answer to a medical or a police need?

Confidentiality: Physicians must maintain patient confidentiality. However, when a physician is a member of a tactical police team, they may be required to disclose information about patients in order to carry out the objectives of the team, especially if the patient being cared for is also the suspect. For example, if the physician discovers that the patient is armed or is carrying an explosive device, care must be interrupted and patient confidentiality must be breached for police to handle the immediate threat [12]. While safety is the number one priority, is important to ensure that patient confidentiality is protected to the greatest extent possible.

Physicians embedded in police tactical teams may allow for improved frontline care. However, it is important to carefully consider the ethical implications of having physicians on tactical police teams and to ensure that their role is clearly defined and consistent with medical ethics. By balancing their medical expertise with their responsibilities as police team members, doctors can play a valuable role in enhancing the safety and well-being of all individuals involved in high-risk law enforcement operations. In-detail knowledge of police tactics and regular tactical training are essential for physicians to assist police tactical teams safely and effectively.

## Data Availability

Not applicable.

## References

[1] Neeki M, DuMontier S, Toy J, Archambeau B, Goralnick E, Pennington T et al. Prehospital Trauma Care in Disasters and Other Mass Casualty Incidents – A Proposal for Hospital-Based Special Medical Response Teams. Cureus [Internet]. 2021 Mar 2 [cited 2023 Apr 20]; Available from: https://www.cureus.com/articles/52887-prehospital-trauma-care-in-disasters-and-other-mass-casualty-incidents---a-proposal-for-hospital-based-special-medical-response-teams. 10.7759/cureus.13657 PMC8016499 33824808

[2] Linger K. Analysis of the Police Response to Mass Shootings in the United States between 1966 and 2016. Emerg Prep Homel Secur Cybersecurity [Internet]. 2018; Available from: https://scholarsarchive.library.albany.edu/honorscollege_ehc/1.

[3] National Association of Emergency Medical Technicians (U.S.), editor. Tactical emergency casualty care (TECC): course manual. Second edition. Burlington, MA: Jones & Bartlett Learning. ; 2020. 136 p.

[4] Smith ER, Shapiro G, Sarani B (2018). Fatal wounding pattern and causes of potentially preventable death following the pulse Night Club shooting event. Prehosp Emerg Care.

[5] Young JB, Sena MJ, Galante JM (2014). Physician roles in Tactical Emergency Medical support: the first 20 years. J Emerg Med.

[6] Service Médical du RAID (2016). Tactical emergency medicine: lessons from Paris marauding terrorist attack. Crit Care Lond Engl.

[7] Colvin L. Tactical Medicine Fellowship | Johns Hopkins Department of Emergency Medicine [Internet]. [cited 2023 Mar 14]. Available from: https://www.hopkinsmedicine.org/emergencymedicine/fellowship_programs/tactical_medicine.html.

[8] Petit NP, Stopyra JP, Padilla RA, Bozeman WP (2019). Resident involvement in Tactical Medicine: 12 years later. Prehospital Disaster Med.

[9] Williams MV, Ajisafe O (2022). How should exposure risk to Tactical Personnel be balanced against clinical and ethical rescue demand?. AMA J Ethics.

[10] Ackerman J (2022). Tactical Emergency Casualty Care and the art of practicing nonmaleficence in Harm’s way. AMA J Ethics.

[11] Aarseth S, Brelin SH, Horn MA, Opsahl JH, Haavardsholm ITØ, Østborg T. Medical ethics in the forced return of migrants. Tidsskr Den Nor Laegeforening Tidsskr Prakt Med Ny Raekke. 2020;140(11). 10.4045/tidsskr.20.0061 32815333

[12] Howell CM, Sontgerath JS, Simonet LB (2016). Unexploded ordnance in an expectant patient: a Case Report. Mil Med.

